# The Present Difficulties of Clinical Pathology

**Published:** 1912-12-21

**Authors:** 


### THE PRESENT DIFFICULTIES OF CLINICAL PATHOLOGY.

### Divided Responsibility or Laboratory Beds?

The clinical laboratory has undoubtedly revolutionised modern hospital practice. The number of cases in which the clinical pathologist is concerned gets larger every week. As diagnosis is the guide to prognosis and treatment, it is imperative that modern hospitals should take every precaution to ensure that laboratory work is done well. To those members of the profession who are fully acquainted with modern laboratory methods it has long been evident that there are many defects in the present arrangements. There is serious reason to doubt whether "clinical pathology" is a legitimate form of specialism. In all other forms of medical specialism the patient is under the care of one man. With the advent of the clinical pathologist we have an entirely new phenomenon. The diagnosis is often made by correspondence between a man who is entirely ignorant of laboratory methods and a second who is ignorant of the clinical features of the case.

### The Errors of Divided Responsibility.

A few instances of the serious errors which can arise from this practice will show that the dangers are not merely hypothetical. The physician or surgeon sees a case of "renal" trouble which he cannot diagnose, and calls in the aid of the clinical pathologist. A specimen of urine is sent to the laboratory, and there comes back a report, "Colon bacillus present." As the urine has probably been passed into the ordinary receptacle, it would be marvellous if there were no colon bacilli present. A catheter specimen properly taken and received in a sterile vessel is, of course, essential, especially if the patient is a female. Again, it is necessary to know whether the urine contains tubercle bacilli. A specimen passed in the ordinary manner is absolutely valueless, for it may contain smegma bacilli, which are almost indistinguishable morphologically from the tubercle bacilli. The present writer knows of a patient who was given a serious prognosis and subjected to a very expensive course of treatment, all owing to the fact that the physician must have been totally ignorant of the elements of bacteriological examination of the urine. An experienced man who knows the ropes will be able to detect these contaminations. He will centrifugalise the urine and, finding vaginal epithelium in the urine, will report it as contaminated, but will probably be thought incompetent for his pains. The average clinician likes his laboratory facts to be gained without any expenditure of time or trouble.

The "Opsonic Index" furnishes further examples of erroneous diagnoses. This suited the phthisiotherapist, for by its means he got cases "early" enough for sanatorium and vaccine treatment to be successful. All he had to do was to take specimens before and after exercise and await the result from the laboratory. One sceptic, however, sent ten or eleven samples of the same serum to different laboratories. The values returned for the serum ranged from subnormal to abnormally high. Hort repeated this experiment, and the results were absolutely unreliable. Had the physicians and surgeons who relied upon the method been acquainted with the technique, they would soon have convinced themselves that the loop-holes for error in opsonic technique were more numerous than even in the clinical diagnosis of pulmonary tuberculosis. Even granting the reliability of Wright's technique and doctrines, it must have been evident to all who applied themselves to the actual technique that very many of the innumerable "opsonists" who sprang up like mushrooms in the night must have been incapable through insufficient training alone.

Clinical chemistry affords many illustrations of the dangers of this divorce between laboratory and clinical workers. A surgeon wished to know whether he might excise a kidney which was excreting through a sinus. He sent up a sample of the urine passed per urethram to know whether it contained a sufficient percentage of urea. There was ample urea in the specimen. By a fortunate accident the specimen was examined by a man who was familiar with renal disease, and the report was endorsed as being absolutely useless as an indication that the other kidney was normal. A week or two later the patient ceased to pass urine per urethram, and all came through the fistula. Had this specimen been sent to the ordinary clinical laboratory, it is quite possible that the surgeon would have been informed that the percentage of urea was normal, and he would have excised the kidney with disastrous results. Obstetricians and surgeons are beginning to have a "little knowledge" of acidosis; and they send up for ammonia-nitrogen co-efficients specimens of urine which are advanced examples of ammoniacal decomposition.

### How the Laboratory Suffers Discredit.

The question of diagnosis of carcinoma of the stomach is one of great importance. Great reliance has been placed on the absence of free hydrochloric acid from the stomach contents. Stomach contents are sent up, and the report comes, "No free hydrochloric acid." The diagnosis of carcinoma is made. As time goes on some of these cases recover, and the laboratory work gets into discredit. If the laboratory examination had been complete, the diagnosis of chronic gastritis, or achylia gastrica, could have been made. The stools should have been examined for "occult blood." Here again the fallacies are obvious to the man who knows both the clinical and the laboratory side. Occult-blood reactions in the absence of free hydrochloric acid from the stomach contents practically spells carcinoma, but it is useless to test for occult blood unless the patient is on a special diet and not taking certain drugs. Again, vomits are sent to the laboratory to be examined for occult blood regardless of the fact that 50 per cent. of all vomits whether due to ulcer or chronic gastritis contain blood. The same applies to stomach contents obtained by passage of the tube; yet a young clinical pathologist recently informed the writer that he obtained much better results for occult blood in gastric contents than in the fæces. Talking to one of the physicians at this same hospital a few days later, one was interested, but not surprised, to hear that the physician did not attach any value to occult-blood tests.

The Divorce between Examiner and Clinician.

These are a few examples which will show what difficulties may arise when we have one man directing treatment on the basis of a second man's examination. The director of treatment is often absolutely ignorant of the means adopted by the laboratory man who has made the diagnosis. Every pathologist who has come into contact with clinicians of the old school knows the incongruities which exist in their minds. Sir Almroth Wright has compared the position indicated above with that of a layman who undertakes to treat a case on the proviso that he shall be able to correspond with some competent medical authority. The fact that the vast majority of physicians and surgeons are laymen when ordinary laboratory technique is the question at issue constitutes the greatest difficulty in the organisation of an efficient laboratory service.

A Via Media Solution.

How can the difficulty be met? The bold way which was adopted by some hospitals in the case of antiseptic surgery, whereby the governing bodies insisted on the surgeons employing antiseptic technique or resigning, is certainly not called for. There seems a very good *via media*. All hospitals should be fitted with a *very good clinical laboratory*. There should be no makeshifts, but all the best labour-saving appliances. There should be good but unqualified laboratory assistants. If this were the case, then any time which the physician or surgeon spent in the laboratory would be well spent. He would be able at least to superintend the work done on his own cases, and would have the opportunity of judging the liability of error. He would keep up to date, and as fresh technique became necessary he would have the opportunity of picking it up. This scheme pre-supposes that some at least of the honorary staff should have had laboratory training. Certainly governing bodies in the future will do well to insist that all candidates for honorary appointments have had laboratory training. A year or two spent almost wholly in a laboratory is a necessary part of the equipment of the modern surgeon and physician.

Beds for Laboratory Cases.

By these means the hospitals would gradually get a staff of well-equipped honoraries who will be able to deal with their individual cases from start to finish. There will be no need for the "clinical pathologist." At present he is neither clinician nor pathologist. He is a sort of compromise, whose chief function is to cover the ignorance of the honorary medical officers. There are many excellent physicians who have never had the opportunity of becoming acquainted with laboratory technique. These men are usually the most pleasant men to work with. They do not disguise their ignorance by much unmeaning talk about toxins, etc., but when laboratory diagnosis is necessary they usually hand over the case altogether to a younger man. It is probable that many of this type would be only too glad to hand over two or three beds to the clinical pathologist, who should have the whole control and responsibility of the cases which required special laboratory examinations. Incidentally, he should also get the credit.

From a fairly extensive experience of hospital work we are convinced that the setting apart of a few beds for "laboratory cases" would be a great advance towards attaining efficiency.

